# Transposon Mutagenesis of Probiotic *Lactobacillus casei* Identifies *asnH*, an Asparagine Synthetase Gene Involved in Its Immune-Activating Capacity

**DOI:** 10.1371/journal.pone.0083876

**Published:** 2014-01-08

**Authors:** Masahiro Ito, Yun-Gi Kim, Hirokazu Tsuji, Takuya Takahashi, Mayumi Kiwaki, Koji Nomoto, Hirofumi Danbara, Nobuhiko Okada

**Affiliations:** 1 Department of Microbiology, School of Pharmacy, Kitasato University, Minato-ku, Tokyo, Japan; 2 Yakult Central Institute for Microbiological Research, Kunitachi, Tokyo, Japan; University of Florida, United States of America

## Abstract

*Lactobacillus casei* ATCC 27139 enhances host innate immunity, and the J1 phage-resistant mutants of this strain lose the activity. A transposon insertion mutant library of *L. casei* ATCC 27139 was constructed, and nine J1 phage-resistant mutants out of them were obtained. Cloning and sequencing analyses identified three independent genes that were disrupted by insertion of the transposon element: *asnH*, encoding asparagine synthetase, and *dnaJ* and *dnaK*, encoding the molecular chaperones DnaJ and DnaK, respectively. Using an *in vivo* mouse model of *Listeria* infection, only *asnH* mutant showed deficiency in their ability to enhance host innate immunity, and complementation of the mutation by introduction of the wild-type *asnH* in the mutant strain recovered the immuno-augmenting activity. AsnH protein exhibited asparagine synthetase activity when the lysozyme-treated cell wall extracts of *L. casei* ATCC 27139 was added as substrate. The *asnH* mutants lost the thick and rigid peptidoglycan features that are characteristic to the wild-type cells, indicating that AsnH of *L. casei* is involved in peptidoglycan biosynthesis. These results indicate that *asnH* is required for the construction of the peptidoglycan composition involved in the immune-activating capacity of *L. casei* ATCC 27139.

## Introduction

Probiotics are defined as live microorganisms which, when administered in sufficient amounts, confer health benefits in the host in addition to their nutritional effects [1]. Among the various health-promoting functions of the probiotics, much attention has been paid to their immune regulatory activity, since they are often used both in clinical applications and as foods that function to maintain the immunologic homeostasis of the host [1], [2].

Lactobacilli are the normal commensals in the human intestines, and certain *Lactobacillus* strains are regarded as probiotic bacteria. *L. casei* strain Shirota has been widely studied for its probiotic effects including immunomodulating activity. It enhances NK cell activity by dairy intake [3], [4] and prevents and reduces the risk of recurrence of bladder cancer by oral administration in human [5], [6]. In addition, this strain also enhances anti-infectious activity against *Listeria monocytogenes* [7], [8] and *Pseudomonas aeruginosa* [9] and inhibits the growth of transplantable allogeneic and syngeneic mouse tumors in several experimental animal models [10]–[14]. However, the detailed molecular mechanisms involved in these processes have not been understood.

*L. casei* ATCC 27139, the parental strain of Shirota [15], has been consistently shown to exhibit anti-infectious activity against *L. monocytogenes* and anti-tumor activity against MethA fibrosarcoma in mice associated with the marked increases in the cytokine levels such as TNF-α, IL-12, IL-18, and IFN-γ [16]. Although *L. casei* ATCC 27139 is susceptible to J1 phage, which shows a remarkably high specificity to certain strains of *L. casei/L. paracasei* [17], J1 phage-resistant mutants of this strain have been reported to be deficient in the immune-activating capacity [16], suggesting that the bacterial components associated with J1 phage sensitivity may be related to the activity. Despite the probable relationship between the J1 phage sensitivity and the immune-activating capacity of this strain, the genetic determinants responsible for J1 phage resistance have not been identified because of the technical limitation of the introduction of mutagenesis using chemical mutagen.

Recently, we developed a random mutagenesis system using a Tn*5*-based EZ::TN transposome for the probiotic *L. casei* ATCC 27139 [18]. Using this system, we constructed a Tn*5* insertion library of nearly 10,000 transposon insertion mutants of *L. casei* ATCC 27139 that represents the first genome-wide random mutagenesis approach for lactobacilli [18]. Analysis of the transposon insertion mutants by Southern hybridization and sequencing of transposon insertion sites confirmed non-biased transposon insertion events [18]. Using a part of the Tn*5* insertion library, we have identified the genes responsible for amino acid auxotrophy, glycine or alanine biosynthesis, respectively, which further allowed the validation of the usefulness of this library [18].

In this study, we describe the identification of the gene required both for the J1 phage sensitivity and the immune-activating capacity of *L. casei* ATCC 27139 using a Tn*5* insertion library. In addition, correlation between probiotic function of *L. casei* ATCC 27139 and cell wall structure was studied.

## Materials and Methods

### Bacterial strains, phage, and growth conditions

*L. casei* ATCC 27139 was used as the wild-type strain. Lactobacilli were cultured in ILS medium [18]. The virulent *L. monocytogenes* strain EGD, was grown in brain heart infusion medium (Becton Dickinson and Co., Franklin Lakes, NJ) prior to infecting the mice. *Escherichia coli* JM109 was used as the host organism for molecular cloning and expression of recombinant proteins. *E. coli* strains were cultured in Luria–Bertani (LB) medium.

A random transposon mutant library was generated using an Tn*5* transposon insertion as described previously [18]. A total of nearly 10,000 mutants were arrayed in a 96-well plate and stored at −80°C in 40% glycerol.

J1 phage was obtained from the American Type Culture Collection (ATCC 27139-B1). After many rounds of phage multiplication in *L. casei* ATCC 27139, a J1 phage lysate was prepared and used for infection as described previously [16], [18]. If required, the following antibiotic concentrations were used: 25 µg/ml of erythromycin for lactobacilli, 500 µg/ml of erythromycin for *E. coli*, 10 µg/ml of chloramphenicol for lactobacilli and *E. coli*, and 100 µg/ml of ampicillin for *E. coli*.

### Plasmid construction

To construct plasmid pYCAT-*asnH* for complementation, *asnH* was amplified by PCR from ATCC 27139 genomic DNA using a primer set of HindIII–*asnH* (5′-CCCAAGCTTCTGGCGCAGAAACATTCTG-3′) and *asnH*–SalI (5′-ACGCGTCGACTCGGCTTGTGGCATCATG-3′). PCR products were digested with HindIII and SalI and subsequently ligated into the same sites of pYCAT, a derivative of the *Escherichia–Lactobacillus* shuttle vector pUCYIT356-1-Not2 [18] carrying the *cat* resistance gene from plasmid pC194 [19].

To construct plasmid expressing an AsnH_WT_-FLAG fusion protein, *asnH* was amplified from ATCC 27139 genomic DNA using a primer set of XhoI–*asnH* (5′-CCGCTCGAGCGGTGCGGAATCATCGCGTTTGC-3′) and XmaI–*asnH* (5′-CCCCCGGGGGTGCTTCAAGTTTCTCTTTAC-3′). PCR products were cloned into the XhoΙ and XmaΙ sites of pFLAG-CTC (Sigma-Aldrich Co., St. Louis, MO), generating plasmid pFLAG-AsnH_WT_. Plasmids expressing AsnH_C2S_-FLAG and AsnH_D265N_-FLAG were created using a QuickChange II site-directed mutagenesis kit (Agilent Technologies, Santa Clara, CA) using a primer set of *asnH*_C2S-Fw (5′-CCGCTCGAGCGGTCCGGAATCATCGCGTTTGC-3′) and *asnH*_C2S-Rv (5′-GCAAACGCGATGATTCCGGACCGCTCGAGCGG-3′) or *asnH*_D265N-Fw (5′-CTTTCATCAGGGGTCAACTCCTCTTATATCACCG-3′) and *asnH*_D265N-Rv (5′-CGGTGATATAAGAGGAGTTGACCCCTGATGAAAG-3′). Mutations were confirmed using BigDye® Terminator v3.1 Cycle Sequencing Kits (Applied Biosystems, Forester City, CA) by DNA sequencing on an ABI PRISM® 3130 Genetic Analyzer (Applied Biosystems).

### Screening of Tn*5* insertion mutants for the J1 phage-resistant phenotype

Tn*5* insertion mutants were screened for their level of J1 phage resistance compared with the wild-type strain as the parental control. Mutants were inoculated into ILS broth containing J1 phage lysate at a multiplicity of infection (moi) of 10 using a 96-well plate. After incubation at 37°C for 48 h, the mutants grown in ILS broth containing J1 phage were regarded as resistant to J1 phage. To ascertain the resistance levels of the mutants to J1 phage infection, the cells of the wild-type strain and the mutants were mixed with J1 phage in ILS broth at an moi of 0.5, and the number of PFU was counted after incubation at 37°C for 24 h. Relative J1 phage sensitivity was calculated as the ratio of PFU for transposon insertion mutants to that of the wild-type strain.

### Analysis of DNA sequence and identification of the transposon insertion site

Self-ligation-mediated inverse PCR was used to characterize the Tn*5* insertion site, as described previously [18]. In brief, NcoI-, BanII-, or KpnI-digested chromosomal DNA from the strain carrying the Tn*5* insertion was self-ligated, and inverse PCRs were performed on ligated templates using a primer set of SqFP (5′-GCCAACGACTACGCACTAGCCAAC-3′) and SqRP (5′-GAGCCAATATGCGAGAACACCCGAGAA-3′). PCR products were sequenced, and the sequences were identified using the BLAST program (http://blast.ncbi.nlm.nih.gov/BLAST.cgi; NCBI, Bethesda, MD).

### Experimental infection

Animal experiments were approved by the Kitasato University Institutional Animal Care and Use Committee (Permit Number: J10-1) and were performed in accordance with the Regulations for the Care and Use of Laboratory Animals in Kitasato University and with the National Research Council Guide for the Care and Use of Laboratory Animals in Japan. Specific pathogen-free male BALB/c mice (Charles River Japan, Kanagawa, Japan) were used for the mouse infection model as described previously [16]. Briefly, the mice were injected intravenously (i.v.) with a heat-killed preparation of lactobacilli (800 µg/mouse) 6 days before *Listeria* infection. The *L. monocytogenes* strain EGD (2.0×10^6^ CFU) was injected i.v. 6 days after lactobacilli inoculation. The number of viable *Listeria* in spleen homogenates 24 h after the challenge was counted; the plates containing the appropriate diluents of the homogenates were maintained at 37°C.

### Cytokine detection by enzyme-linked immunosorbent assay (ELISA)

Heat-killed lactobacilli preparations (800 µg) were injected i.v. into mice. IL-12, TNF-α, and IFN-γ were measured from spleen homogenates 8 h after the challenge using OptEIA™ ELISA kits (BD Biosciences Pharmingen, San Diego, CA) as described by the manufacturer. Absorbances were measured using a model 3550 microplate reader (Bio-Rad Laboratories, Hercules, CA). Sample data were extrapolated from standard curves using Microplate Manager® 6 software (Bio-Rad Laboratories).

### Expression and purification of recombinant proteins

*E. coli* BL21 (TaKaRa Bio, Shiga, Japan) strains containing plasmids encoding FLAG fusion proteins were grown at 37°C in LB broth overnight. The cultures were diluted 1∶100 into fresh LB broth supplemented with 0.4% (w/v) glucose and incubated at 25°C for 3 h. Isopropyl-b-d-thiogalactopyranoside was added to a final concentration of 1 mM, and the cultures were further incubated at 25°C for 5 h. To purify FLAG fusion proteins, bacteria were centrifuged at 7000×*g* for 10 min at 4°C and resuspended in 10 ml of CelLytic B™ reagent (Sigma-Aldrich Co.) with cOmplete, Mini protease inhibitor cocktail tablets (Roche Diagnostics Co., Basel, Switzerland). After a 15 min incubation at room temperature, samples were centrifuged at 15,000×*g* for 20 min at 4°C to pellet cellular debris. FLAG fusion proteins were purified using an anti-FLAG M2 affinity gel according to manufacturer's instructions (Sigma-Aldrich Co.). Protein concentrations were quantified according to the Bradford method using the protein assay dye reagent concentrate (Bio-Rad Laboratories).

### Enzymatic activity of AsnH

The enzymatic activity of AsnH was measured using an Amplex® Red Glutamic Acid/Glutamate Oxidase Assay Kit (Molecular Probes, Eugene, OR) according to the methods described previously [20]. Briefly, aspartic acid or cell wall extracts of *L. casei* ATCC 27139, used as the substrates, were diluted with a reaction mixture containing 50 mM Tris-HCl (pH 7.5), 15 mM MgCl_2_, 25 mM KCl, 1 mM glutamine, 0.2 mM dithiothreitol, and 2 mM ATP. Several concentrations of glutamic acid (0–20 µM) were used as the standard. Fifty microliters of the substrate solution were added to 50 µl of the Amplex® Red reagent/horse radish peroxidase/glutamate oxidase/glutamate–pyruvate transaminase/alanine working solution. After incubation at 30°C for 30 min, luminescence was detected using a SpectraMax5 microplate reader (Molecular Devices Co., Sunnyvale, CA) with excitation at 544 nm and emission at 595 nm. Sample data were extrapolated from standard curves using Microplate Manager® 6 software (Bio-Rad Laboratories).

### Gram staining and transmission electron microscopy

Gram staining of *L. casei* cells grown to the early stationary phase in ILS broth was performed using Fiber G (Nissui Pharmaceutical Co.), in accordance with the manufacturer's recommendations, and the cells were observed using OLYMPUS BX50 light microscope (OLYMPUS Co., Tokyo, Japan). For transmission electron microscopy (TEM) analyses, *L. casei* cells were fixed with 2% glutaraldehyde in 100 mM phosphate buffer (pH 7.4), postfixed in osmium tetroxide at 4°C, dehydrated, and embedded in Epon 812 resin. Ultrathin sections were analyzed using JEM-1200EX TEM (JEOL, Tokyo, Japan) at 80 kV.

### Preparation of cell wall extracts

Two-hundred-milliliter cultures of *L. casei* grown to the early stationary phase in ILS broth were centrifuged at 8000×*g* at 4°C for 10 min. The cells were washed 3 times with distilled water and resuspended in 20 ml of acetone. Suspensions were centrifuged at 3000×*g* at 4°C for 10 min, and precipitates were washed again with acetone and suspended in 10 ml of chloroform/methanol (2∶1). Suspensions were incubated at 60°C for 30 min and centrifuged at 3000×*g* at 4°C for 10 min. Precipitates were resuspended in 50 mM Tris-HCl (pH 7.0) with DNaseI and RNaseA at final concentrations of 1000 U and 50 ng/µl, respectively, and incubated at 37°C for 1 h. Proteinase K was added to the lysates to a final concentration of 1 mg/ml, and suspensions were heated at 37°C for 16 h. After centrifugation at 20,000×*g* at 4°C for 40 min, precipitates were washed with 50 mM Tris-HCl (pH 7.0) and treated with 0.1% (w/v) sodium dodecyl sulfate in distilled water at 100°C for 10 min. Suspensions were then centrifuged at 20,000×*g* at 4°C for 40 min, and precipitates were washed with distilled water 6 times. Precipitates were then washed with 20% (v/v) ethanol and 5% (w/v) NaCl 3 times and again with distilled water 3 times. After centrifugation at 20,000×*g* at 4°C for 40 min, precipitates were lyophilized and used as cell wall extracts.

### Amino acid composition of the peptidoglycans

Cell wall extracts prepared as described above were suspended in 5 ml of 10% (v/v) trichloroacetic acid and heated at 100°C for 20 min. After centrifugation at 12,000×*g* at 4°C for 30 min, precipitates were washed 3 times with 50 mM phosphate buffer (pH 7.2) and then with 100% (v/v) ethanol. The resultant residues were suspended with diethyl ether and centrifuged at 15,000×*g* at 4°C for 10 min. Precipitates were lyophilized and used as the peptidoglycan fraction. The purified peptidoglycans (1 mg) were hydrolyzed in 6 N HCl at 100°C for 16 h, and the amino acid composition was analyzed by high-performance liquid chromatography using a Waters 2695 Separations Module (Waters co., Milford, MA) composed of a C_18_ AccQ·Tag column, a 474 scanning fluorescence detector, and AccQ (all from Waters co.).

### Sensitivity to lysozyme

To ascertain the sensitivity of *L. casei* strains to lysozyme, *L. casei* ATCC 27139 and its mutants that had been pre-incubated in ILS broth at 37°C for 48 h were inoculated at 1.0×10^7^ CFU/ml into fresh ILS broth containing various lysozyme concentrations (0–10,000 µg/ml). Following incubation at 37°C for 48 h, the minimal inhibitory concentration of lysozyme at which *L. casei* strains failed to grow was regarded as its MIC.

### Statistical analysis

The results of peptidoglycan thickness are presented as the means ± standard error of the mean (SEM) and the others are presented as the means ± standard deviations (SD) of triplicate experiments. Statistical analyses were performed using Student's *t*-test. Differences were considered significant at *p*<0.05.

### Nucleotide sequence accession number

The sequence orthologs of the *asnH* gene were obtained from the DDBJ/EMBL/GenBank database. The accession numbers for the *Rhodococcus erythropolis*, *Corynebacterium glutamicum*, *Mycobacterium smegmatis*, and *Escherichia coli* sequences were AB183824, BX842579, AB183824, J05554, respectively, and GeneID of the *Lactococcus lactis* was 4797108. The alignment was constructed using the GENETYX program (Genetyx Co., Tokyo, Japan).

## Results

### Isolation of J1 phage-resistant mutants of *L. casei* ATCC 27139 from a random Tn*5* insertion library

To identify genes involved in the augmentation of host innate immunity by *L. casei*, a total number of 9408 Tn*5* insertion mutants from the mutant library were screened for their J1 phage sensitivity using a 96-well plate assay. Nine mutants (MT77, MT2890, MT3332, MT4156, MT5740, MT6674, MT7279, MT7398, and MT8105) displayed significantly higher resistance to J1 phage compared with the wild-type parental strain. Sensitivities to J1 phage infection of these mutants were 10^4^-fold lower than that of the wild-type strain (Table 1). Southern hybridization analyses confirmed that all these mutants carried a single independent Tn*5* transposon insertion (data not shown).

**Table 1:** Summary of relative sensitivities to J1 phage infection and predicted transposon insertion loci of J1 phage-resistant mutant.

Strain		Relative J1 phage sensitivity^a^	Predicted transposon insertion gene^b^
*L. casei*	ATCC 27139	1.0	–
	MT77	4.4×10^−5^	asparagine synthetase (Glutamine-hydrolyzing) *asnH*
	MT2890	7.5×10^−5^	chaperone protein *dnaJ*
	MT3332	1.3×10^−4^	chaperone protein *dnaJ*
	MT4156	7.5×10^−5^	asparagine synthetase (Glutamine-hydrolyzing) *asnH*
	MT5740	9.1×10^−5^	chaperone protein *dnaJ*
	MT6674	9.7×10^−5^	chaperone protein *dnaJ*
	MT7279	9.2×10^−5^	chaperone protein *dnaK*
	MT7398	9.6×10^−5^	chaperone protein *dnaJ*
	MT8105	6.0×10^−5^	asparagine synthetase (Glutamine-hydrolyzing) *asnH*

^a^ The cells of *L. casei* ATCC 27139 and isogenic Tn*5* insertion mutants (1.0×10^7^ CFU/ml) were combined with J1 phage (5.0×10^6^ PFU/ml) in ILS broth at an moi of 0.5, and PFU were counted after incubation at 37°C for 24 h. The relative sensitivities to J1 phage were assessed as the ratio of PFU for transposon insertion mutants to that of the wild-type strain.

^b^ The genes into which transposons were inserted were identified using BLAST.

Inverse PCR and sequencing of the DNA regions flanking the transposon insertion sites of each J1 phage-resistant mutant indicated that *asnH*, *dnaJ*, and *dnaK* were disrupted by insertion of the transposon element: among the 9 Tn*5* mutants, 3 mutants (MT77, MT4156, and MT8105) harbored Tn*5* insertions in *asnH*, 5 mutants (MT2890, MT3332, MT5740, MT6674, and MT7398) carried insertions in *dnaJ*, and the remaining mutant (MT7279) carried a transposon insertion in *dnaK*.

### The *asnH* mutant of *L. casei* ATCC 27139, but not the *dnaJ* or *dnak* mutants, is deficient in the immune-augmenting activity

**Figure 1:**
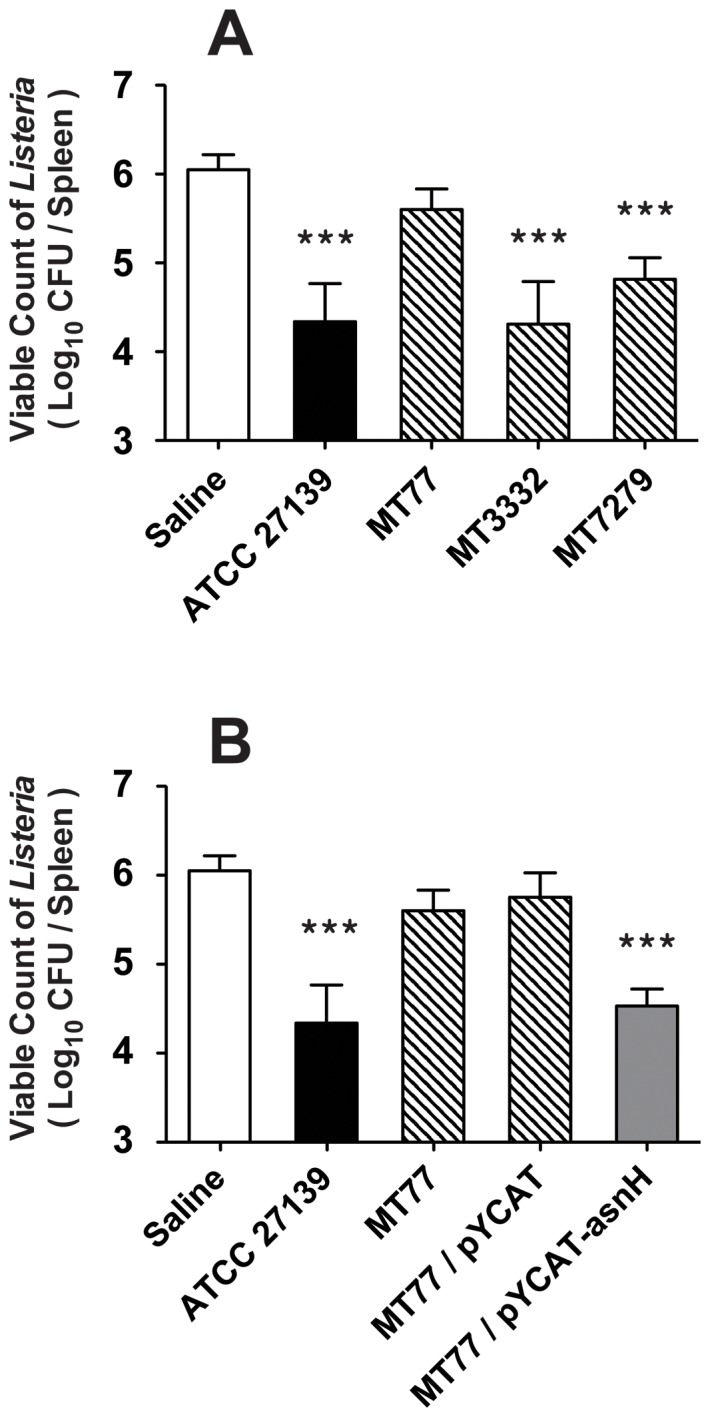
Anti-listerial activity of J1 phage-resistant Tn*5* insertion mutants of *L. casei*. Heat-killed preparations of *L. casei* ATCC 27139 or isogenic Tn*5* insertion mutants (800 µg) were injected i.v. into BALB/c mice 6 days before an i.v. challenge with *L. monocytogenes* (2.0×10^6^ CFU/mouse). Six mice per group were dissected 24 h after the challenge, and viable *Listeria* were detected in spleens. Columns: white, saline; black, *L. casei* wild-type; slashed, *L. casei* J1 phage-resistant mutant; gray, *L. casei asnH* mutant complemented with the cloned *asnH*. Results are depicted as the means ± standard deviations (SD). Statistical significance was calculated using the Student's *t*-test. Significant differences indicated between the control and treated groups. ***, *p*<0.001.

**Figure 2:**
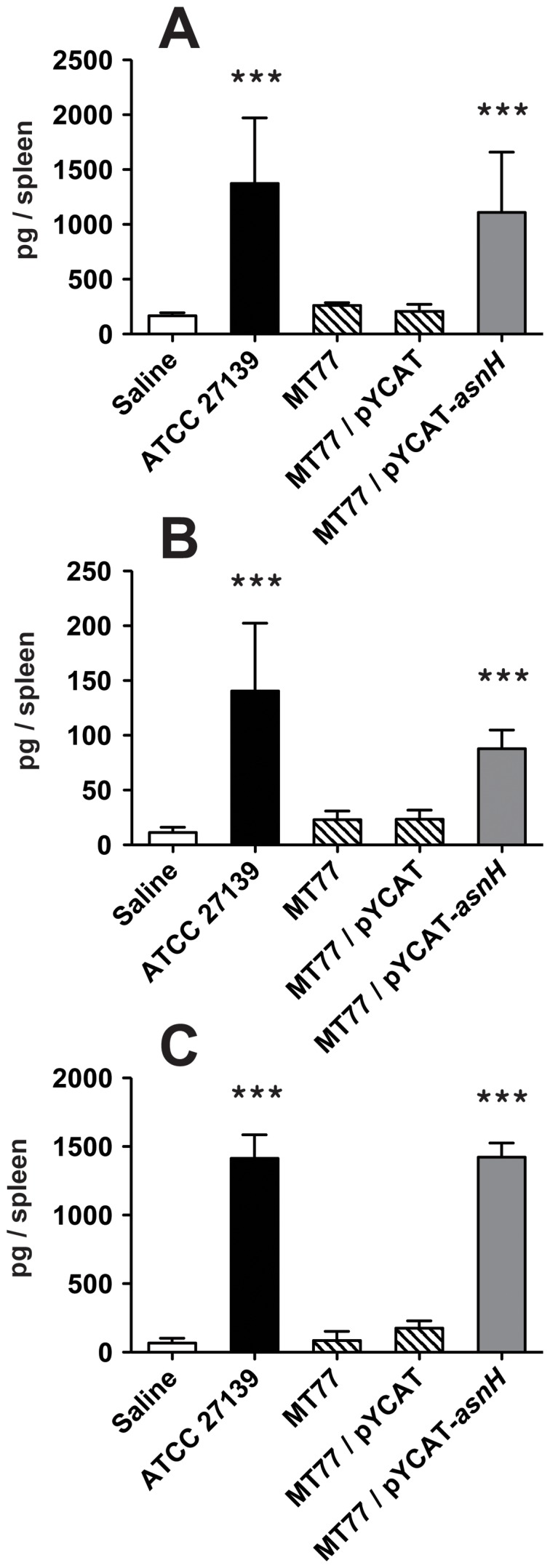
Induction of cytokine in spleen after *L. casei* administration. Heat-killed preparations of *L. casei* ATCC 27139 or isogenic Tn*5* insertion mutants (800 µg) were injected i.v. into BALB/c mice. Six mice per group were sacrificed 8 h after injection, and IL-12 (A), TNF-α (B), and IFN-γ (C) proteins were measured from spleen homogenates by ELISA. Columns: white, saline; black, *L. casei* wild-type; slashed, *L. casei* J1 phage-resistant mutant; gray, *L. casei asnH* mutant complemented with the cloned *asnH*. Results are depicted as the means ± standard deviations (SD). Statistical significance was calculated using the Student's *t*-test. Significant differences indicated between the control and treated groups. ***, *p*<0.001.

Since previous studies have demonstrated that the heat-killed preparation of J1 phage-resistant mutants of *L. casei* ATCC 27139 are deficient in the immune-augmenting activity, we assessed this activity in the J1 phage-resistant Tn*5* mutants obtained in this study using a mouse model of *Listeria* infection. Whereas mice pre-treated with *L. casei dnaJ* (MT3332) and *dnak* (MT7279) mutant strains exhibited anti-listerial activity comparable to that of the wild-type strain, the *asnH* mutant (MT77) showed no significant activity (Fig. 1A). The defect in anti-listerial activity in the *asnH* mutant resulted solely from the absence of *asnH* because significant protection against *Listeria* infection in mice could be restored to wild-type levels by introducing a plasmid harboring *asnH* into the mutant (Fig. 1B). As for the induction of innate cytokines such as IL-12, TNF-α, and IFN-γ in the host, the *asnH* mutant did not induce significant levels of cytokines, and complementation of the *asnH* mutant with a plasmid expressing intact AsnH restored the cytokine production comparable to that of the wild-type strain (Fig. 2).

### AsnH is an asparagine synthetase with glutamine amidotransferase activity

**Figure 3:**
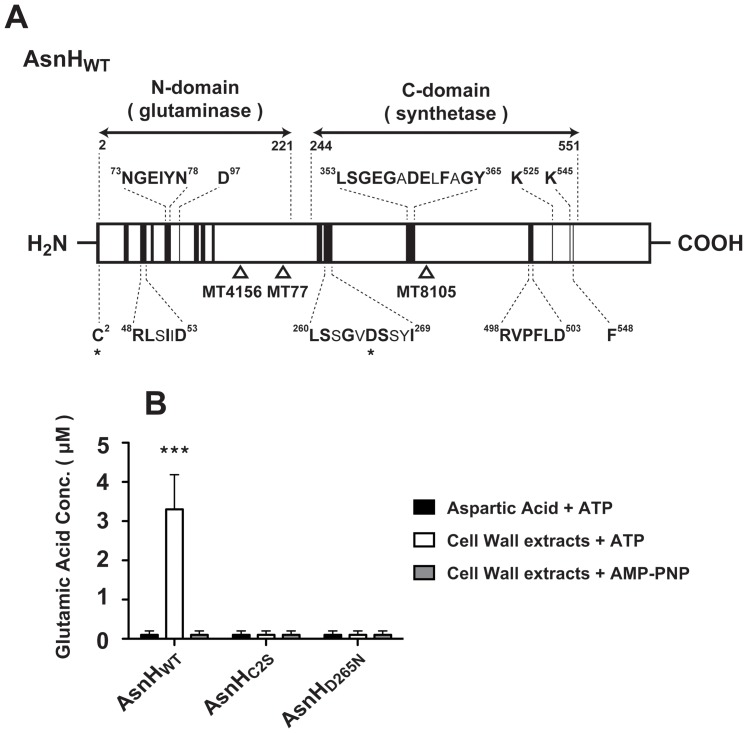
Schematic diagram of *L. casei* AsnH and glutaminase activity of AsnH proteins. (A) Protein sequence alignment was performed using the GENETYX program. Solid boxes indicate highly conserved regions. Regions containing residues important for enzymatic activities are shown in detail (identical residues are indicated by boldface type). The first methionine was consistently included in the numbering and was designated Met-1. Asterisks indicate mutated residues in this study that were critical to glutaminase or synthetase activities. (B) Recombinant AsnH_WT_, mutant AsnH_C2S_, and mutant AsnH_D265N_ (0.1 µg) were examined using the glutaminase activity test. Five microliters of a reaction mixture (initial volume, 50 µl) were quenched at 30 min to estimate glutamic acid concentration. Glutaminase activity was monitored in the presence of aspartic acid or cell wall extracts of ATCC 27139, ATP or AMP–PNP plus cell wall extracts. Results are depicted as the means ± standard deviations (SD). Statistical significance was calculated using the Student's *t*-test. Significant differences indicated between the control and treated groups. ***, *p*<0.001.

*asnH* (1,878 bp) of *L. casei* ATCC 27139 encodes a 625-amino acid product with a predicted molecular mass of 73 kDa. A homology search determined that the *asnH* product has significant homology to the LtsA/AsnB glutamine amidotransferase family of proteins that have been identified in various Gram-positive and Gram-negative bacteria. The glutamine amidotransferase family includes proteins composed of an N-terminal glutaminase domain and a C-terminal synthetase domain with conserved amino acid motifs [20], [21]. In AsnH of *L. casei* ATCC 27139, amino acid residues Cys-2, Arg-48, Asn-73, Glu-75, and Asp-97, which are essential for glutaminase activity of the N-terminal domain, and amino acid residues Ser-261, Asp-265, Ser-266, Gly-355, Asp-359, Lys-525, and Lys-545, which are required for catalytic activity of the C-terminal domain, are invariant (Fig. 3A) [20], [22]–[24].

To determine whether AsnH possess glutamine amidotransferase activity, we conducted an *in vitro* glutamic acid production assay. Glutaminase activity can be enhanced by the addition of an amide acceptor such as aspartic acid for *E. coli* AsnB or lysozyme-treated cell wall extracts for *Rhodococcus erythropolis* LtsA [20]. Incubation of the purified AsnH_WT_-FLAG fusion proteins with glutamine resulted in glutamic acid production at a rate of 6.6 µM/h when lysozyme-treated cell wall extracts of *L. casei* ATCC 27139, but not aspartic acid, was added in the reaction mixture (Fig. 3B). To further characterize the AsnH as a glutamine amidotransferase, we used site-directed mutagenesis to replace the conserved N-terminal Cys-2 with Ser-2 and the conserved C-terminal Asp-265, which is located in the putative ATP-binding pocket of the synthetase domain, with Asn-265. The AsnH_C2S_-FLAG and AsnH_D265N_-FLAG mutant proteins failed to produce glutamic acid (Fig. 3B). In AsnB of *E. coli*, ATP and aspartic acid form a catalytic intermediate and NH_3_ is transferred to the amide acceptor [21]. To test whether the enzymatic activity of AsnH of *L. casei* is enhanced by ATP hydrolysis, a non-hydrolyzable ATP analog, AMP–PNP [adenosine 5′-(βγ-imido) triphosphate], was used instead of ATP in the reaction. No glutamic acid production was detected in the AMP–PNP reaction mixture (Fig. 3B).

### Structural analysis of the *asnH* mutant cell wall

**Figure 4:**
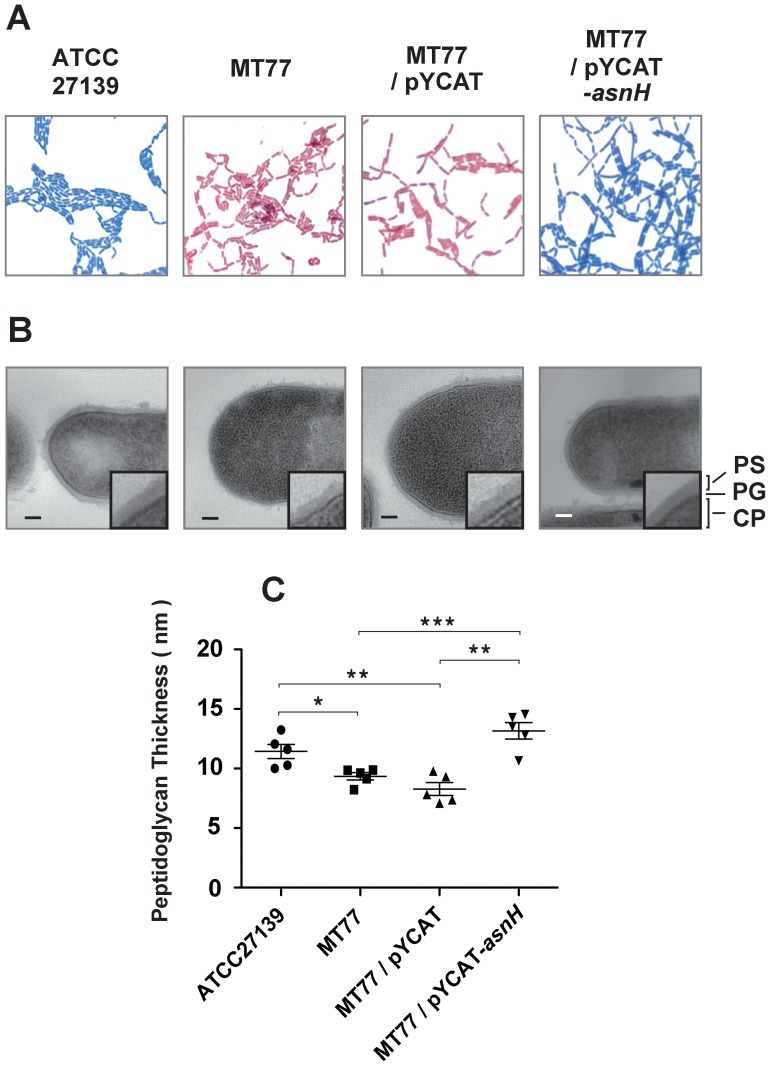
Micrographs of *L. casei* cells. (A) Gram staining was performed using Fiber G. The cells were observed under a light microscope (×1000). (B) Representative micrographs indicating cell morphologies in several different fields. Digitally magnified (×3) views of typical cell surfaces are shown in the insets. Scale bars = 100 nm. PS, polysaccharide; PG, peptidoglycan; CP, cytoplasm. (C) Peptidoglycan thickness of *L. casei* cells are presented as the means ± standard error of the mean (SEM) (n = 5). Statistical significance was calculated using the Student's *t*-test. *, *p*<0.05; **, *p*<0.01; ***, *p*<0.001.

AsnH of *Lactococcus lactis* and LtsA of *R. erythropolis* are involved in cell wall biosynthesis, and the mutations in *asnH* or *ltsA* confer increased bacterial sensitivity to lysozyme [20], [25]. *L. casei* wild-type and *asnH*-complemented mutant strains were stained Gram positive, while the *asnH* mutant (MT77) was stained Gram negative (Fig. 4A). TEM observations of *L. casei* cells showed that the peptidoglycan layers of the mutant cells were significantly thinner (Fig. 4BC) and the cell size appeared to be larger when compared with those of the wild-type and the complemented cells (Fig. 4B).

**Figure 5:**
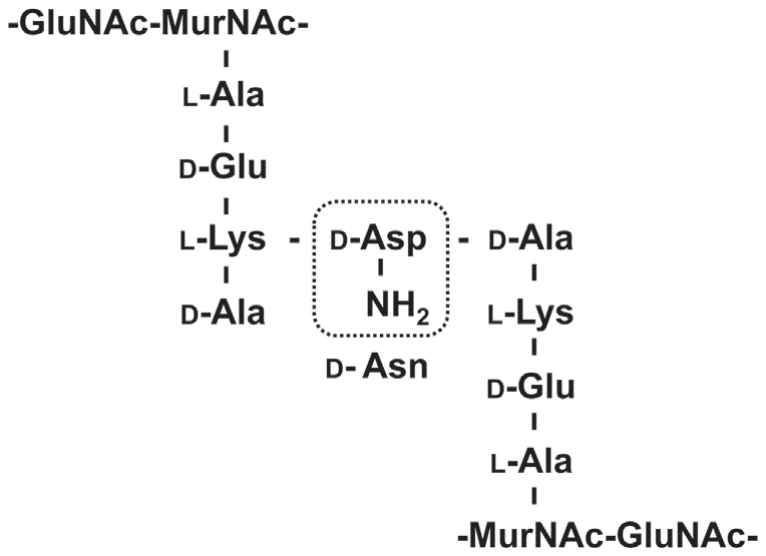
Schematic representation of disaccharide tetrapeptide with crossbridge in peptidoglycan of *L. casei*. GluNAc: *N*-acetyl glucosamine, MurNAc: *N*-acetyl muramic acid, L-Ala: L-alanine, D-Glu: D-glutamic acid, L-Lys: L-lysine, D-Ala: D-alanine, D-Asp: D-aspartic acid, D-Asn: D-asparagine.

In *L. casei*, the peptidoglycan stem peptide is composed of the tetrapeptide chain containing l-Ala-d-Glu-d-Lys-d-Ala, which is cross-linked with a d-Asp residue (Fig. 5) [26]. To evaluate the effect of *asnH* mutation on the cell wall structure, we analyzed the amino acid composition of the peptidoglycan layers of the *L. casei* cells. The molar ratio of Asp in the peptidoglycans extracted from the *asnH* mutant strain was lower than that of extracts from the wild-type and the complemented strains (Table 2).

**Table 2:** Amino acid compositions in peptidoglycans and lysozyme MICs of J1 phage-resistant mutants.

		Molar ratio in peptidoglycan^a^				
Strain		Ala	Glu	Lys	Asp	Lysozyme MIC^b^ (µg/ml)
*L. casei*	ATCC 27139	1.25	1	0.69	0.96	>10,000
	MT77	1.53	1	0.67	0.65	80
	MT77/pYCAT	1.56	1	0.66	0.55	80
	MT77/pYCAT-*asnH*	1.26	1	0.67	0.93	>10,000

^a^ Amino acid compositions in peptidoglycans were analyzed by high-performance liquid chromatography after samples had been hydrolyzed for 16 h with 6 M HCl at 100°C. Assays were performed in two independent experiments, and the differences of each molar ratio between experiments were less than 10%.

^b^ The cells of *L. casei* ATCC 27139 and isogenic Tn*5* insertion mutants (1.0×10^7^ CFU/ml) were inoculated into fresh ILS broth containing several concentrations of lysozyme. After incubation at 37°C for 48 h, the minimal inhibitory concentration of lysozyme at which *L. casei* strains failed to grow was regarded as its MIC.

The lysozyme MIC of the *asnH* mutant (MT77) was 80 µg/ml, which is far lower than that of the wild-type strain as: >10,000 µg/ml (Table 2). The *asnH*-complemented strain displayed a lysozyme resistance comparable to the wild-type strain (Table 2). The vancomycin MIC of the *asnH* mutant was 8-fold lower than that of the wild-type and the complemented strains (data not shown).

### Identification of the mutated region of MNNG-induced J1 phage-resistant mutants

**Figure 6:**
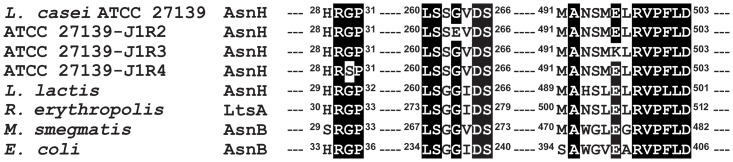
Partial sequence alignment of *L. casei* AsnH and orthologs. Partial sequences were selected from highly conserved regions of the following *L. casei* AsnH orthologs: *L. lactis*, *R. erythropolis*, *M. smegmatis*, and *E. coli*. A black background indicates identical residues in all bacteria.

We previously demonstrated that J1 phage-resistant mutants, which were obtained by chemical mutagenization of *L. casei* ATCC 27139 with *N*-methyl-*N*′-nitro-*N*-nitrosoguanidine (MNNG), exhibited the lower immune-augmenting activity [27]. To further clarify that AsnH is implicated in the activity, we cloned *asnH* from the MNNG-induced J1 phage-resistant mutants (strains 27139-J1R1, 27139-J1R2, 27139-J1R3, and 27139-J1R4). The mutation sites in the genes were found to be either a deletion (C-1207 deletion in 27139-J1R1) or displacement (G-788 to A-788 in J1R2, G-1486 to A-1486 in J1R3, and G-88 to A-88 in J1R4) of a base in *asnH*, resulting in frameshift (J1R1) or missense mutations (Gly-263 to Glu-263 in J1R2, Glu-496 to Lys-496 in J1R3, and Gly-30 to Ser-30 in J1R4) of AsnH (Fig. 6). These mutated residues are among the evolutionarily conserved amino acids essential for enzymatic activity of members of the LtsA/AsnB glutamine amidotransferase family (Fig. 6). Introducing a plasmid harboring *asnH* into the MNNG-induced J1 phage-resistant mutant strains recovered the J1 phage-sensitive phenotype (Table 3) and restored the immunoprotective effects associated with *Listeria* infection in mice (data not shown).

**Table 3:** Relative sensitivities to J1 phage infection among MNNG-induced J1 phage-resistant mutants.

		Relative J1 phage sensitivity		
		Plasmid		
Strain		–	/pYCAT	/pYCAT-*asnH*
*L. casei*	ATCC 27139	1.0	NT	NT
	MT77	4.4×10^−5^	4.9×10^−5^	1.1
	J1R	1.2×10^−4^	3.0×10^−4^	0.8
	J1R2	1.1×10^−4^	1.4×10^−4^	1.1
	J1R3	1.1×10^−4^	2.1×10^−4^	1.1
	J1R4	1.4×10^−4^	8.4×10^−5^	0.8

The relative sensitivities to J1 phage were assessed as the ratio of PFU for MNNG-induced mutants to that of the wild-type strain. NT, not tested.

## Discussion

Heat-killed *L. casei* ATCC 27139 significantly augments innate immunity in mice [16], [27]. This immunoprotective effect is lost in J1 phage-resistant mutants [16], [27], suggesting that certain cell wall components associated with J1 phage sensitivity are involved in the activation of host innate immunity. In this study, we used a Tn*5* insertion library to demonstrate that *asnH*, *dnaJ*, and *dnaK* are coordinated with J1 phage sensitivity, and only *asnH* mutants lost the ability to enhance host innate immunity. Complementation of the *asnH* mutant strains with the cloned *asnH* recovered the activity. MNNG-induced J1 phage-resistant mutants lacking the capacity to augment innate immunity all had mutated *asnH*, reinforcing the idea that *asnH* is essential for the activity of *L. casei* ATCC 27139 to boost host innate immunity.

DnaJ and DnaK proteins are molecular chaperones that regulate protein folding and function as heat shock proteins [28]. Because the *dnaJ* and *dnaK* mutants of *L. casei* ATCC 27139 displayed anti-listerial activity equivalent to the wild-type strain, it is unlikely that the *dnaJ* or *dnaK* gene contributes to the immune-augmenting activity. Loss of innate immunity-augmenting activity in J1 phage-resistant mutants does not result simply from the phage resistance but from a specific phenomenon related to the loss of AsnH activity.

Asparagine synthetase, which consists of glutaminase activity and synthetase activity, catalyzes asparagine and glutamic acid biosynthesis from aspartic acid and glutamine, respectively [29], [30]. The synthetase activity of the C-terminal domain recognizes an amide donor and forms an intermediate before the glutaminase activity of the N-terminal domain hydrolyzes an amide donor, and this involves an ATP-dependent transfer of ammonia to aspartic acid yielding asparagine in the presence of magnesium ion [31]. Although glutamine-hydrolyzing AsnB of several bacteria utilizes glutamine as an amide donor [32], [33] and glutaminase activity is markedly enhanced when the amide acceptor aspartic acid is added in the reaction mixture [24], glutaminase activity of *R. erythropolis* LtsA is enhanced significantly when lysozyme-treated cell wall extracts are added instead of aspartic acid in the reaction mixture [20]. In the present study, *L. casei* ATCC 27139 AsnH displayed marked glutaminase activity when the lysozyme-treated cell wall extracts of ATCC 27139 were added instead of aspartic acid, strongly suggesting that components of the lysozyme-treated cell wall extracts serve as amide acceptors. *L. lactis* AsnH, which exhibits 59% amino acid sequence identity with the *L. casei* AsnH, is involved in amidation of d-Asp present in the interpeptide cross-bridge of peptidoglycan (Fig. 5) [25]. In the maturation of *L. lactis* peptidoglycan, approximately 75% of the side chain and cross-bridge residues are amidated, indicating that d-Asp is converted to d-Asn [34]. Homologous genes have been identified in other Gram-positive bacterial species that possess d-Asn in their cross-bridges, and *L. casei* peptidoglycan also possesses d-Asn in its cross-bridges [35]. In this study, the assays of the *asnH*-mutant cells about lysozyme sensitivity, Gram staining, and TEM observations showed the lack of the thick and rigid peptidoglycan features that are characteristic to the wild-type cells, indicating that AsnH is required for the synthesis of intact peptidoglycans. Sequence analysis by SignalP [36] indicates that AsnH contains neither a putative signal peptide nor a transmembrane segment, suggesting that AsnH is localized inside the bacterial cytoplasm and that this protein most probably acts on peptidoglycan precursors. Although the analysis of amino acid compositions in peptidoglycan of the *asnH* mutant cells showed a decrease in aspartic acid content, further experiments would be needed to elucidate where AsnH protein acts on the peptidoglycan. Peptidoglycan and derived muropeptides in *L. salivarius* showed protective effect in murine colitis mice model through induction of pathogen-associated molecular pattern receptors such as Nod2 [37], which recognizes intracellular muramyl dipeptide of a peptidoglycan constituent [38], suggesting the relevance of peptidoglycan structure to the immune-augmenting activity of *L. casei* ATCC 27139.

On the other hand, the changed amino acid compositions in peptidoglycan may affect the immune signaling by non-peptidoglycan molecules. Cell surface polysaccharide components, d-galactosamine and l-rhamnose, in *L. casei* ATCC 27139/S-1 comprise a phage receptor for J1 phage, and J1 phage-resistant mutant lacked d-galactosamine in its surface components [39]. In this study, although no significant difference in the polysaccharide thickness between the wild-type and the *asnH* mutant cells has been identified (data not shown), the amount of polysaccharide in the *asnH* mutant cells appeared to be less organized. In *L. casei* strain Shirota, polysaccharide-peptidoglycan complexes play important roles in augmenting host innate immunity [7], [40], which, taken together, suggests the relevance of cell surface polysaccharide components to the immune-activating capacity of *L. casei* ATCC 27139. Teichoic acids in *L. plantarum* has been shown to modulate intestinal and systemic immune responses in a TLR2-dependent manner [41]. TLR2 recognizes bacterial components such as lipoteichoic acid, lipoprotein [42], [43], LPS [44], [45], the mycobacterial lipoarabinomannan [46], and heat-killed Gram-positive bacteria [47]. *L. casei* ATCC 27139-J1R1, *asnH* mutant, lost the capacity to induce TLR2 and Nod2 [16]. Demonstration of the differences in the amount of cell wall components between the wild-type and the *asnH* mutant cells might lead to further elucidation of the components and structures that enhance host innate immunity.

In conclusion, *asnH* of the probiotic *L. casei* ATCC 27139 is suggested to be involved in the immune-activating capacity of this strain. *asnH* contributes to peptide synthesis in the peptidoglycan layer, indicating that certain cell wall structures of *L. casei* are important in the enhancement of host innate immunity. These findings should facilitate the characterization of active components and structures in probiotic lactobacilli to augment host innate immunity.
